# Assessment of the methodological quality of studies on core outcome sets for respiratory diseases: A systematic review and meta-research study

**DOI:** 10.1371/journal.pone.0316670

**Published:** 2025-01-02

**Authors:** Mengjuan Liu, Jiajia Wang, Lu Wang, Xinyi Zhang, Ruiyu Hao, Duolao Wang, Tao Chen, Jiansheng Li

**Affiliations:** 1 Collaborative Innovation Center for Chinese Medicine and Respiratory Diseases Co-constructed by Henan Province & Education Ministry of P.R. China/Henan Key Laboratory of Chinese Medicine for Respiratory Diseases, Henan University of Chinese Medicine, Zhengzhou, China; 2 Department of Respiratory Diseases, The First Affiliated Hospital of Henan University of Chinese Medicine, Zhengzhou, China; 3 The First Clinical Medical School, Henan University of Chinese Medicine, Zhengzhou, China; 4 Department of Clinical Sciences, Liverpool School of Tropical Medicine, Liverpool, United Kingdom; Azienda Ospedaliera Universitaria SS Antonio e Biagio e Cesare Arrigo, Alessandria, University of Eastern Pedemont, ITALY

## Abstract

**Background:**

With increasing attention to core outcome sets (COS), the number of studies on COS for respiratory diseases (COS-RD) is on the rise. However, the methodological quality is still unclear. Therefore, we conducted a study to assess the methodological quality of studies on COS-RD.

**Methods:**

PubMed, Embase, Cochrane Library, and Web of Science were searched for study protocols or original studies on COS-RD about adults, from their inception to February 23, 2024. The COMET database and Chinese databases (including China National Knowledge Infrastructure, Wanfang Data, Chongqing VIP database, and China Biology Medicine) were also searched as a supplement. Two researchers independently screened the literature, extracted the data, and assessed the methodological quality of included studies using the Core Outcome Set-STAndardised Protocol (COS-STAP) statement, the Core Outcome Set-STAndards for Development (COS-STAD) recommendations, and the Core Outcome Set-STAndards for Reporting (COS-STAR) statement.

**Results:**

A total of 27 articles (five study protocols and 22 original studies, 26 studies) were included in this study. For the assessment of study protocols using the COS-STAP statement, the item with the lowest complete reporting rate was "missing data" (Item 9, 40.0%), while "description how outcomes may be dropped/combined, with reasons" (Item 5b, 60.0%) and "dissemination" (Item 11, 60.0%) had relatively low complete reporting rates. For the assessment of original studies using the COS-STAD recommendations, the item with the highest non-reporting rate was "care was taken to avoid ambiguity of language used in the list of outcomes" (Item 11, 45.5%), while "the population(s) covered by COS" (Item 3, 31.8%) and "the intervention(s) covered by COS" (Item 4, 31.8%) had relatively high non-reporting rate. When using the COS-STAR statement to assess the original studies, the item with the lowest complete reporting rate was "protocol deviations" (Item 11, 13.6%), while “describe how outcomes were dropped/combined, with reasons (if applicable)” (Item 6b, 36.4%), "participants" (Item 5, 40.9%), "ethics and consent" (Item 10, 54.5%), "protocol/registry entry" (Item 14, 63.6%), and “outcome scoring” (Item 8, 63.6%) had relatively low complete reporting rates.

**Conclusion:**

The methodological quality of studies on COS-RD needs to be further improved. The appropriate use of aforementioned international reporting standards can advance the methodological quality and reporting transparency of studies on COS-RD.

## Introduction

Core outcome sets (COS) refer to an agreed-upon standard set of outcomes that should be measured and reported, as a minimum, in all clinical trials in specific areas of health or healthcare, so as to improve the usefulness of outcomes and reduce the heterogeneity between different clinical trial outcomes [1]. Currently, an increasing number of COS are being applied in clinical studies and practice, and the Core Outcome Measures in Effectiveness Trials (COMET) database has included 370 published COS, covering 18 disease categories, including cancer, rheumatology, neurology, heart & circulation, lungs & airways, and others [2]. One research has shown that COS is not typically used and/or reported in late phase trials, which may hinder the evaluation of intervention effects and evidence synthesis, leading to a waste of research resources [3]. There are several factors that limit the application of COS, such as the poor quality and design of some COS, the use was trialist’s own outcome preferences and choice, and the preference for researchers to select their own outcomes [3]. To enhance the standardization of research reporting, the COMET working group released the Core Outcome Set-STAndards for Reporting (COS-STAR) statement in 2016 [4]. In 2018, the COMET working group published the Core Outcome Set-STAndards for Development (COS-STAD) recommendations, which outline 11 minimum standards that need to be met during the development process of a COS [5]. These standards are designed to help COS developers design research projects and assist COS users in assessing the methodological quality of developed COS. Subsequently, the COMET working group released the Core Outcome Set-STAndardised Protocol (COS-STAP) statement [6]. The difference among the above three is that the COS-STAR statement relates to the reporting of COS development studies, the COS-STAD recommendations focus on the principles of design associated with COS development, while the COS-STAP statement focuses on the report of a COS development study protocol. The introduction of these international reporting standards is conducive to enhancing the completeness, transparency, and quality of studies, and making it easier for COS users to assess the value and applicability of COS.

Respiratory diseases are common and prevalent, and some of them seriously endanger human health, such as coronavirus disease 2019 (COVID-19), chronic obstructive pulmonary disease (COPD), lung cancer, asthma, etc. In recent years, significant progress has been made in clinical studies around these diseases, and the number of studies on COS for respiratory diseases (COS-RD) is gradually increasing [2,7]. However, there was currently no systematic analysis on the methodological quality of COS-RD. Therefore, the purpose of this meta-research study was to assess the methodological quality of studies on COS-RD using the COS-STAP statement, COS-STAD recommendations, and COS-STAR statement.

## Materials and methods

### Inclusion and exclusion criteria

The inclusion criteria included one of the following: (i) COS-RD about adults, (ii) study protocols on COS-RD; or (iii) original studies reporting the COS-RD. The exclusion criteria included any of the following: (i) studies only involving intermediate steps, such as systematic reviews, Delphi methods, and so on; (ii) COS studies only focusing on children; (iii) COS methodological studies; (iv) repeated articles; or (v) articles whose full texts could not be obtained.

### Search strategy

PubMed, Embase, Cochrane Library, and Web of Science were searched for the studies on COS-RD from their inception to February 23, 2024. The databases were searched using free text term "core outcome set*" in the field of "title" or "abstract". The COMET database and Chinese databases, including China National Knowledge Infrastructure (CNKI), Wanfang Data, Chongqing VIP Database (VIP), and China Biology Medicine (CBM), were also searched as a supplement. In addition, the reference lists of potentially eligible studies and relevant systematic reviews [8–10] were reviewed. We developed detailed search strategies for each electronic database without language restrictions. The detailed search strategies are provided in S2 Table.

### Literature selection and data extraction

Two investigators independently screened the literature and extracted the data, and disagreements were resolved by consulting a third investigator. All the retrieved literature was imported into the EndNote Version X9.2 (Thomson ResearchSoft, Stanford, CA, USA) and the duplicate records were removed. The title and abstract of the literature were reviewed first to exclude irrelevant ones, then the full text was reviewed to determine eligible ones. Data extraction was carried out using a pre-defined form. The form contained the characteristics of the included studies, such as title, authors, year of publication, target disease, registration number, study type, research scope, research methods used, and stakeholders.

### Quality assessment

The COS-STAR statement [4] and COS-STAD recommendations [5] were used to assess the reporting quality of the included original studies, and the COS-STAP statement [6] was used to assess the reporting quality of the included study protocols. Accordingly, the report compliance of each item was judged as "fully reported", "partially reported" or "not reported". “Fully reported” refers to all the elements included in the item being fully reported with each element being clearly described, “partially reported” refers to the elements included in the item being partially reported, or individual elements not being clearly described, while “not reported” refers to none of the elements included in the item being reported. Two researchers independently scored the COS studies. If there was a disagreement, a third person would be asked for consultation.

### Statistical analysis

The SPSS Version 26.0 (IBM Corp, Armonk, NY, USA) was used to calculate the frequency and proportion of "fully reported", "partially reported", and "not reported " of each item in the COS-STAR statement [4], COS-STAD recommendations [5], and COS-STAP statement [6]. The GraphPad Prism Version 9.5.1 (GraphPad Software, Boston, MA, USA) was used for graphical plotting.

## Results

### Study selection

**Fig 1:**
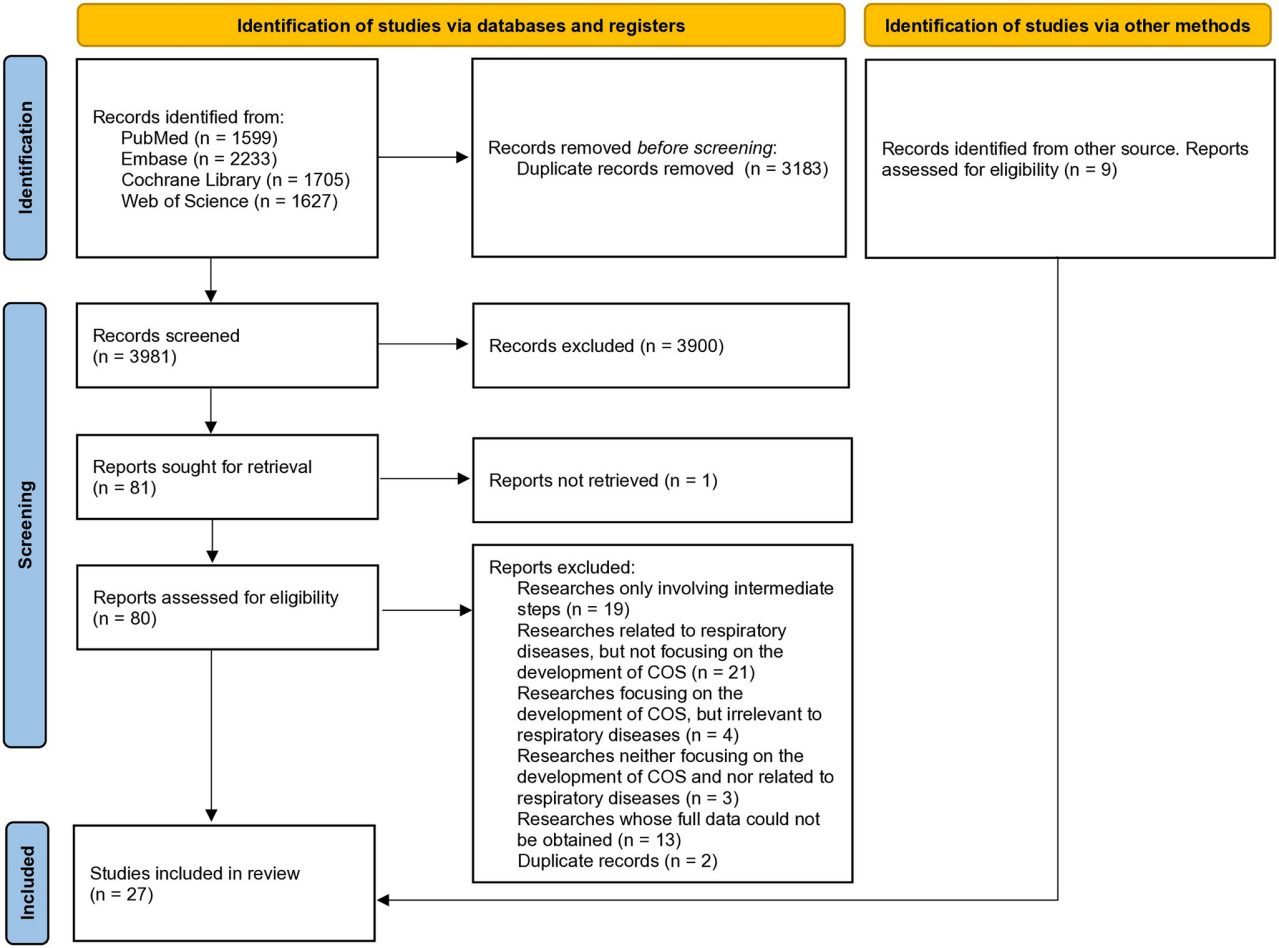
Flow diagram of study selection.

A total of 7164 records were retrieved from PubMed, Embase, Cochrane Library, and Web of Science, and 3981 records were retained after removing the duplicate ones. Then 3900 records were excluded due to ineligibility by reviewing the titles and abstracts. 62 records were further excluded by reading the full text, and 18 articles met the eligibility criteria. In addition, 9 articles were identified from other sources and included. Finally, 27 articles [11–37] (five study protocols and 22 original studies, 26 studies) were included, involving COVID-19 (eight articles) [11–18], COPD (six articles) [19–24], lung cancer (five articles) [29–33], asthma (two articles) [36,37], bronchiectasis (two articles) [27,28], pulmonary sarcoidosis (two articles) [34,35], chronic pulmonary heart disease (CPHD) (one article) [25], and obstructive sleep apnea hypopnea syndrome (OSAHS) (one article) [26]. All the 26 studies included were registered on the COMET website (https://comet-initiative.org/), and only one study published both study protocol [21] and original study [22]. The selection procedure is illustrated in Fig 1. Detailed characteristics of included studies are presented in Table 1.

**Table 1:** Characteristics of included studies.

Registration number	References	Type	Scope				Stakeholder	Research method
			Health condition	Population	Intervention	Research or practice setting		
1548	Tong 2020[11]	Result	COVID-19	People with confirmed or suspected COVID-19	Unreported	Clinical research	①②③④⑤⑫⑮	ADE
1848	Tong 2021[12]	Result	COVID-19	People with confirmed or suspected COVID-19	Unreported	Clinical research	①②③④⑤⑫⑮	E
1523	Jin 2020[13]	Result	COVID-19	Patients from mild and ordinary to severe and critical types, and rehabilitation period	Different interventions (either pharmaceutical or non-pharmaceutical therapies)	Clinical trials, systematic reviews/meta-analyses, guidelines, and other research on evidence evaluation and decision-making	①③⑥⑧⑨⑩⑫	ADE
1507	Qiu 2020[14]	Result	COVID-19	Patients with confirmed COVID-19 cases of “mild”, “ordinary”, “severe”, or “critical” types	Traditional Chinese medicine and Western medicine	Randomized controlled trials and observational studies	①②③④⑤⑥⑨	ADE
1528	Marshall 2020[15]	Result	COVID-19	Patients from asymptomatic viremia to complete recovery or death	Unreported	Clinical research	①③④⑫⑮	AB
2332	Munblit 2022[16]	Result	COVID-19	Post-COVID-19 condition in adults (≥18 years of age)	Unreported	Clinical research and practice settings	①③④⑤⑥⑦	ACDE
1847	Gorst 2023[17]	Result	COVID-19	Post-COVID-19 condition in adults (≥18 years of age)	Unreported	Clinical research	①③④⑥⑦	ACDE
1810	Shepherd 2022[18]	Result	COVID-19	People living in care homes	Pharmacological and non-pharmacological interventions for preventing COVID-19 infection and transmission	Clinical research	①③④⑤⑫⑬	ACDE
1151	Souto-Miranda 2023[19]	Result	COPD	Unreported	Pulmonary rehabilitation	Clinical research	①②③④⑫	ABDE
1826	Camus-García, 2021[20]	Result	COPD	Adults with COPD	Self-management interventions	Clinical research	①②③④	ADE
1325	Mathioudakis 2020[21]	Protocol	AECOPD	Adults with COPD exacerbations of any severity	All interventions.	Clinical research, focusing on RCTS	①②③④⑪⑫⑮	ABDE
1325	Mathioudakis 2022[22]	Result	AECOPD	Unreported	Pharmacological and nonpharmacological interventions	Clinical research	①②③④⑥⑦⑪⑫⑬	ABDE
2232	Verburg 2019[23]	Result	COPD	People in the Netherlands:	Primary care physical therapy	Clinical research	①③④⑫⑮	ABE
1744	Zhao 2022[24]	Result	COPD	Unreported	Traditional Chinese medicine	Clinical research	①⑥⑩⑪⑫	ABDE
1677	Niu 2021[25]	Protocol	CPHD	Unreported	Traditional Chinese medicine	Clinical research	①③④⑥⑫⑬⑭	ABDE
1544	Wanyan 2020[26]	Protocol	OSAHS	Adults with OSAHS	All interventions.	Clinical research	①②③④⑫⑬	ABDE
1931	Hamzeh 2022[27]	Protocol	Bronchiectasis	Bronchiectasis in adults	All physiotherapy interventions, including airway clearance, positive expiratory pressure devices, and pulmonary rehabilitation	Physiotherapy effectiveness trials	①③④	ABDE
936	Spargo 2019[28]	Result	Bronchiectasis	Bronchiectasis in adults	All interventions for the long-term management	Clinical research	①②③④⑮	ABD
2086	Edbrooke 2023[29]	Protocol	lung cancer	Patients with non-small cell and small cell lung cancer at any stage who are older than 18 years old	Exercise or physical activity rehabilitation interventions (supervised and/or unsupervised)	Clinical research	①③④⑤⑬	ABDE
2477	de Rooij 2022[30]	Result	lung cancer	Unreported	Immunotherapy and targeted therapy,	Clinical research	①②③④⑪⑫	ADE
2163	Escudero-Vilaplana 2020[31]	Result	lung cancer	Newly diagnosed lung cancer (including non-small cell and small cell lung cancer) patients in Spain	All interventions	Clinical research	①④⑫	ABE
1141	Mak 2016[32]	Result	lung cancer	Newly diagnosed lung cancer (including non-small cell and small cell lung cancer) patients	Therapeutic measures aimed at cure or palliation (including optimal supportive care)".	Clinical research	①②④	AD
1483	Li 2021[33]	Result	lung cancer	Unreported	Traditional Chinese medicine	Clinical research	①③④⑥⑩⑪⑫⑬⑭	ABDE
1156	Harman 2022[34]	Result	Pulmonary sarcoidosis	Unreported	All interventions	Clinical research	①③④⑪⑫⑬⑮	ABDE
1318	Kampstra 2019[35]	Result	Pulmonary sarcoidosis	Unreported	Unreported	Clinical research	①②③	AD
1353	Tejwani 2021[36]	Result	Asthma	Patients with moderate-to-severe asthma in adults and children aged greater than or equal to 5 years	Pharmacological interventions	Phase 3 and 4 clinical drug trials	①③④⑪⑫⑮	ABDE
1698	Khaleva 2023[37]	Result	Asthma	Severe asthma patients in Paediatric (children and adolescents aged 6–17 years) and adult (≥18 years)	Biological therapies	Clinical research	①②③④⑥⑪⑫	ABDE

Note: ①Clinical doctor; ②Nurse; ③Clinical researcher; ④Patient/Caregiver; ⑤Public; ⑥Evidence/Methodology Expert; ⑦Clinical Epidemiologist; ⑧Pharmacologist; ⑨Statistician; ⑩Journal Editor; ⑪Pharmaceutical Company Representative; ⑫Policy Makers/Government Representatives/Healthcare Administrators/Healthcare Decision Makers; ⑬COS Developers; ⑭COS Users; ⑮Sponsors; A: Systematic Review; B: Qualitative Research; C: Extracting Other Relevant COS; D: Delphi Survey; E: Consensus Meeting.

### Quality assessment of included studies

#### The results of quality assessment using COS-STAP statement

**Fig 2:**
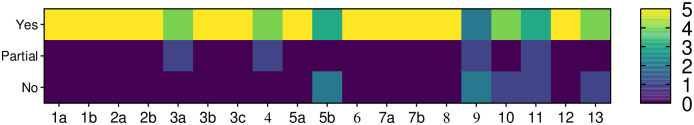
The results of quality assessment using COS-STAP statement. Note: Detailed item information is shown in S3 Table.

All of the five study protocols fully reported “title” (Item 1a), “abstract” (Item 1b), “background and objectives” (Item 2a and item 2b), “described the intervention(s) that will be covered by the COS” (Item 3b), and “context of use for which the COS was to be applied” (Item 3c). One protocol [25] partially reported “the health condition(s) and population(s) that will be covered by the COS” (Item 3a, 20.0%). Two protocols [25,27] fully reported “missing data” (Item 9, 40.0%), and this item had the lowest reporting rate. The full reporting rates were also low for “description how outcomes may be dropped/combined, with reasons” (Item 5b, 60.0%) and “dissemination” (Item 11, 60.0%). Additionally, two protocols [21,27] did not report “describe how outcomes may be dropped/combined, with reasons” (Item 5b, 40.0%), and two protocols [21,29] did not report “describe how missing data will be handled during the consensus process” (Item 9, 40.0%). The quality assessment results of study protocols are shown in Fig 2 and S3 Table.

#### The results of quality assessment using COS-STAD recommendations

**Fig 3:**
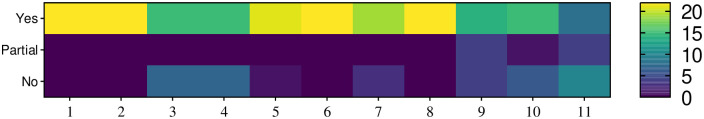
The results of quality assessment using COS-STAD recommendations. Note: Detailed item information is shown in S4 Table.

All of the 22 original studies fully reported “the research or practice setting(s) in which the COS is to be applied” (Item 1), “the health condition(s) covered by the COS” (Item 2), “healthcare professionals with experience of patients with the condition” (Item 6), and “the initial list of outcomes considered both healthcare professionals’ and patients’ views” (Item 8). Seven studies [19,22,24,30,33–35] did not report “the population(s) covered by the COS” (Item 3, 31.8%), and seven studies [11,12,15–17,22,35] did not report “the intervention(s) covered by the COS” (Item 4, 31.8%) in the scope specification domain. In the stakeholders involved domain, three studies [13,15,35] did not report “patients with the condition or their representatives” (Item 7, 13.6%), and one study [32] did not report “those who will use the COS in research” (Item 5, 4.5%). In the domain of consensus process, only eight studies [14,19,20,22,28,30,34,37] focused on “care was taken to avoid ambiguity of language used in the list of outcomes” (Item 11, 45.5%), which had the highest non-reporting rate. The non-reporting rates for "criteria for including/dropping/adding outcomes were described a priori" (Item 10) and "a scoring process and consensus definition were described a priori" (Item 9) were 27.3% and 18.2%, respectively. The results of quality assessment using COS-STAD recommendations are shown in Fig 3 and S4 Table.

#### The results of quality assessment using COS-STAR statement

**Fig 4:**
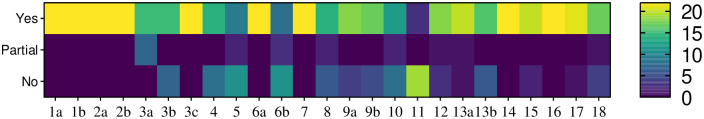
The results of quality assessment using COS-STAR statement. Note: Detailed item information is shown in S5 Table.

In the domain of title, abstract, and introduction, seven studies [19,22,24,30,33–35] partially reported item 3a, seven studies [11,12,15–17,22,35] did not report item 3b, while all studies fully reported the remaining items (Item 1a, item 1b, item 2a, item 2b, and item 3c). In the domain of methods, all original studies fully described the information sources used to identify an initial list of outcomes (Item 6a) and how the consensus process was undertaken (Item 7). The item with the lowest complete reporting rate was "describe how outcomes were dropped/combined, with reasons (if applicable)" (Item 6b, 36.4%). 11 studies [11,12,15,24,28,30–34,36] did not report “participants” (Item 5, 50.0%). Eight studies [11,12,15,23,31,32,35,36] did not report “protocol/registry entry” (Item 4, 36.4%), and eight studies [11,12,15,22,24,32,35,36] did not report “ethics and consent” (Item 10, 36.4%). In the domain of results, all original studies fully listed the outcomes in the final COS (Item 14). “protocol deviations” had the lowest fully reporting rate (Item 11, 13.6%). The non-reporting rates for "describe any new outcomes introduced and any outcomes dropped, with reasons, during the consensus process" (Item 13b), “present data on the number and relevant characteristics of the people involved at all stages of cos development” (Item 12) and "list all outcomes considered at the start of the consensus process" (Item 13a) were 72.7%, 81.8% and 90.9%. In the discussion domain, all original studies provided full interpretations of the final COS in the context of other evidence, and implications for future research (Item 16). two studies [11,15] did not report “limitations” (Item 15, 9.1%). In the other information domain, four studies [19,20,24,33] did not report “conflicts of interest” (Item 18, 18.2%), and one study [24] did not report “funding” (Item 17, 4.5%). The results of quality assessment using COS-STAR statement are shown in Fig 4 and S5 Table.

## Discussion

This study systematically reviewed the published COS study protocols and original studies and assessed the methodological quality of these studies utilizing the COS-STAR statement, COS-STAD recommendations, and COS-STAP statement. The assessment results indicated that only three studies [14,20,28] fully reported all COS-STAD items, with the majority of studies on COS-RD not meeting the international reporting standards. It is recommended that future efforts may focus on the following aspects.

First of all, it is found that there is a notable difference in the number of COS established among various respiratory diseases. For example, only one COS [25] was developed for CPHD, while eight COS [11–18] were particularly designed for COVID-19. This may be due to that the COVID-19 epidemic has triggered a global emergency, drawing additional attention to the development of COS for COVID-19. In contrast, certain illnesses like CPHD may not get as much funding or attention. More high-quality studies on COS for respiratory disorders not limited to those mentioned in this study is still needed in the future. Additionally, the differences in the included outcome measures between COS for the same disease were also found. For example, Jin et al. [13] developed a COS for COVID-19 involving 12 outcome measures across the mild type, ordinary type, severe type, critical type, and rehabilitation period. By comparison, Qiu et al. [14] developed a COS for COVID-19 consisting of 17 outcome measures across clinical outcomes, etiology, inflammatory factors, vital signs, blood and lymphatic-system parameters, respiratory outcomes, clinical efficacy, and symptoms. The two COS involve different populations, interventions, and research settings. The reasons for these discrepancies may be attributed to the various scopes of COS. To improve the specificity of COS and facilitate users to select COS that applies to their needs, the COS developers should provide detailed descriptions of the target population and interventions involved. For instance, the description should clarify whether COS covers all patients with a certain disease (e.g., lung cancer) or a specific subgroup (e.g., non-small cell lung cancer) and whether the interventions covered by COS encompass all beneficial interventions for the disease or are specific to certain types of interventions, such as surgery, medication, or medical devices [5]. Additionally, we would recommend developing or updating a COS if significant limitations prevent it from meeting current research needs. For example, a study suggested that due to the lack of patient input into the current research, the emergence of new patient-reported outcomes, and improved understanding of the pathophysiology of juvenile idiopathic arthritis, the existing research for juvenile idiopathic arthritis needs to be revised [38].

Stakeholders primarily include trialists, health service users, health care practitioners, regulators, industry representatives, policymakers, researchers, patients, and the public [39]. Many studies on COS-RD have underreported or inadequately reported details such as the number, expertise, geographical distribution of stakeholders, and rationale for their involvement in COS development. The number and professional composition of representatives from each stakeholder group have an impact on the scientific and practical nature of COS. Therefore, it is recommended to describe how stakeholder groups are selected, including the specific number of individuals in each group, their qualifications, and the selection methods employed [4]. During the development of three COS [13,15,35], relevant disease patients and their representatives were not included. A study [35] on COS for pulmonary sarcoidosis pointed out that during its online webinar, only consensus was reached among all members of the expert group, without incorporating the viewpoints of patients, which may lead to potential bias. The involvement of patients is crucial in the development of COS. A study has shown that involving patients in the development of COS increases the likelihood of incorporating outcomes that reflect the impact on their daily lives [40]. As experienced experts, their contributions complement the knowledge of scientists and professionals [41]. However, patients may exhibit differences in how they perceive the disease and assess the results of treatments due to variances in age, cognitive function, educational background, and socioeconomic status, etc. This may result in variations in the outcomes that are chosen. In the future, we should pay attention to assessing patient representativeness [42] and emphasizing patient education during participation [43,44]. Additionally, patients may participate to the COS development process through various methods such as interviews, focus groups, and surveys, etc. but the optimal methods for including patients still need to be considered [45].

Although the registration information for COS was not directly reported in eight studies [11,12,15,23,31,32,35,36], it was discovered that the included studies were registered on the website by manually searching the COMET website and comparing and verifying the original texts. To improve access to the most recent information on relevant COS and limit the likelihood of duplicate COS development, future studies should include full reporting of COS registration information. At present, only one study on COS for COPD has published both protocol and original study. This is not conducive to restricting the behavior of not reporting or selectively reporting protocol changes after the start of the study, and it hinders the transparency and completeness of the research [4]. Three studies [16,22,30] reported deviations from specific protocols. A systematic review of COS for obstetrics and gynecology found that none of the studies met the item, and further analysis is needed to explore whether COS that does not deviate from the protocol should report this item [46]. A small number of studies have underreported or not reported the methods of disseminating COS. When developing COS, it is important to provide detailed explanations of how the COS will be disseminated. This may include descriptions of dissemination through journal publications, conference presentations, research websites, and relevant associations to expand its dissemination [6]. Chevance et al. [47] suggested that increasing the number and diversity of stakeholders involved in development may also enhance the uptake of COSs in trials.

In addition, only two study protocols [25,27] fully reported on missing data in the consensus process. Personalised reminder emails to participants (with details of current response rates), personalised emails from distinguished researchers in the field, and direct telephone calls have been found to be helpful strategies in reducing the potential for missing data to occur [39]. Most studies did not focus on the language description of the outcome list. To reduce the occurrence of language bias, the COS developers should make the language easy to be understood. Among the four studies [19,20,24,33] with omitted conflict of interest information, three were master’s theses. The reason for the omission of these items is likely related to the lack of requirements for reporting conflicts of interest in master’s theses.

Interestingly, with the increasing global attention to traditional Chinese medicine (TCM), a complementary and alternative medicine, there is a growing number of TCM-related COS [48,49]. In the current study, four studies [14,24,25,33] reported TCM-related COS-RD, two of which reported the TCM syndrome score as a core outcome [24,33]. The TCM syndrome refers to a pathological summarization on the disease location, etiological factors, nature, severity, and prognosis in a certain stage [50]. The TCM-related COS lacks the outcomes reflecting the characteristic of TCM, and the TCM syndrome score has not received sufficient attention [49]. Generally speaking, studies on TCM-related COS including COS-RD still need to be explored.

This study is not without limitations. Although we searched relevant databases and traced potentially eligible studies, the possibility of missing potential studies can not be completely eliminated. Additionally, some of the included studies [23,28,32,35] began before the publication of the international reporting standards, making it impossible for them to adhere to the standards in their research design. However, the main purpose of this study is to provide references for future high-quality studies by assessing the methodological quality of current studies instead of criticizing quality of existing COS, thus getting around this restriction. Finally, we did not succeed to register the protocol, which may be a potential limitation.

## Conclusions

The majority of studies on COS-RD do not comply with international reporting standards, and the methodological quality of studies on COS-RD requires further improvement, including the following areas: providing a complete report on the scope of COS and stakeholder information, increasing patient involvement, and focusing on the publication study protocols on COS-RD. When developing a COS, researchers may be recommended to prioritize the COS-STAR statement, COS-STAD recommendations, and COS-STAP statement. At the same time, they should fully understand the meaning of each item. To avoid wasting research resources, relevant databases should be checked before starting a new COS study. If no COS exists, a new one may be developed. If an existing COS has significant limitations, the revision and update of the existing research should be considered.

## Supporting information

S1 Table PRISMA 2020 checklist. (DOCX)

S2 Table Details of the literature search strategy. (DOCX)

S3 Table Compliance with COS-STAP items. (DOCX)

S4 Table Compliance with COS-STAD items. (DOCX)

S5 Table Compliance with COS-STAR items. (DOCX)

S6 Table Literature list. (XLSX)
