# Supplementary material for: Assessment of the methodological quality of studies on core outcome sets for respiratory diseases: A systematic review and meta-research study
Source: PLoS One. 2025 Jan 2;20(1):e0316670. doi: 10.1371/journal.pone.0316670 (PMC11695018; doi:10.1371/journal.pone.0316670)
Supplement: S2 Table — (DOCX) [file pone.0316670.s002.docx]

**S2 Table. Details of the literature search strategy.**

| **Search** | **Query** | **Items found** |
| --- | --- | --- |
| **PubMed** | | |
| #1 | core outcome set*[Title/Abstract] | 1599 |
| **Embase** | | |
| #1 | 'core outcome set*':ti OR 'core outcome set*':ab | 2233 |
| **Cochrane Library** | | |
| #1 | (core outcome set*):ti OR (core outcome set*):ab | 1705 |
| [**Web of Science**](https://apps.webofknowledge.com/home.do?SID=6BQQjiiMCVa9MgFvRpC) **core collection** | | |
| #1 | TI=("core outcome set*") OR AB=("core outcome set*") | 1627 |
