# Supplementary material for: Assessment of the methodological quality of studies on core outcome sets for respiratory diseases: A systematic review and meta-research study
Source: PLoS One. 2025 Jan 2;20(1):e0316670. doi: 10.1371/journal.pone.0316670 (PMC11695018; doi:10.1371/journal.pone.0316670)
Supplement: S3 Table — (DOCX) [file pone.0316670.s003.docx]

**S3 Table. Compliance with COS-STAP Items.**

| **SECTION/TOPIC** | **Item**  **No.** | **CHECKLIST ITEM** | **Mathioudakis 2020[21]** | **Niu 2021[25]** | **Wanyan 2020[26]** | **Hamzeh 2022[27]]** | **Edbrooke 2023[29]** |
| --- | --- | --- | --- | --- | --- | --- | --- |
| **TITLE/ABSTRACT** |  |  |  |  |  |  |  |
| Title | 1a | Identify in the title that the paper describes the protocol for the planned development of a COS | Y | Y | Y | Y | Y |
| Abstract | 1b | Provide a structured abstract | Y | Y | Y | Y | Y |
| **INTRODUCTION** |  |  |  |  |  |  |  |
| Background and objectives | 2a | Describe the background and explain the rationale for developing the COS, and identify the reasons why a COS is needed and the potential barriers to its implementation | Y | Y | Y | Y | Y |
|  | 2b | Describe the specific objectives with reference to developing a COS | Y | Y | Y | Y | Y |
| Scope | 3a | Describe the health condition(s) and population(s) that will be covered by the COS | Y | P | Y | Y | Y |
|  | 3b | Describe the intervention(s) that will be covered by the COS | Y | Y | Y | Y | Y |
|  | 3c | Describe the context of use for which the COS is to be applied | Y | Y | Y | Y | Y |
| **METHODS** |  |  |  |  |  |  |  |
| Stakeholders | 4 | Describe the stakeholder groups to be involved in the COS development process, the nature of and rationale for their involvement and also how the individuals will be identified; this should cover involvement both as members of the research team and as participants in the study | Y | P | Y | Y | Y |
| Information sources | 5a | Describe the information sources that will be used to identify the list of outcomes. Outline the methods or reference other protocols/papers | Y | Y | Y | Y | Y |
|  | 5b | Describe how outcomes may be dropped/combined, with reasons | N | Y | Y | N | Y |
| Consensus process | 6 | Describe the plans for how the consensus process will be undertaken | Y | Y | Y | Y | Y |
| Consensus definition | 7a | Describe the consensus definition | Y | Y | Y | Y | Y |
|  | 7b | Describe the procedure for determining how outcomes will be added/combined/dropped from consideration during the consensus process | Y | Y | Y | Y | Y |
| **ANALYSIS** |  |  |  |  |  |  |  |
| Outcome scoring/  feedback | 8 | Describe how outcomes will be scored and summarised, describe how participants will receive feedback during the consensus process | Y | Y | Y | Y | Y |
| Missing data | 9 | Describe how missing data will be handled during the consensus process | N | Y | P | Y | N |
| **ETHICS and DISSEMINATION** |  |  |  |  |  |  |  |
| Ethics approval/  informed consent | 10 | Describe any plans for obtaining research ethics committee/institutional review board approval in relation to the consensus process and describe how informed consent will be obtained (if relevant) | N | Y | Y | Y | Y |
| Dissemination | 11 | Describe any plans to communicate the results to study participants and COS users, inclusive of methods and timing of dissemination | P | N | Y | Y | Y |
| **ADMINISTRATIVE INFORMATION** |  |  |  |  |  |  |  |
| Funders | 12 | Describe sources of funding, role of funders | Y | Y | Y | Y | Y |
| Conflicts of interest | 13 | Describe any potential conflicts of interest within interest the study team and how they will be managed | Y | Y | Y | N | Y |

Note: N, no (=not reported); P, partly (=partially reported); Y, yes (= fully reported).
