# Supplementary material for: Assessment of the methodological quality of studies on core outcome sets for respiratory diseases: A systematic review and meta-research study
Source: PLoS One. 2025 Jan 2;20(1):e0316670. doi: 10.1371/journal.pone.0316670 (PMC11695018; doi:10.1371/journal.pone.0316670)
Supplement: S4 Table — (DOCX) [file pone.0316670.s004.docx]

**S4 Table. Compliance with COS-STAD Items.**

| **Domain** | **Standard number** | **Methodology** | **Tong 2020[11]** | **Tong 2021[12]** | **Jin 2020[13]** | **Qiu 2020[14]** | **Marshall 2020[15]** | **Munblit 2022[16]** | **Gorst 2023[17]** | **Shepherd 2022[18]** | **Souto-Miranda 2023[19]** | **Camus-García 2021[20]** | **Mathioudakis 2022[22]** | **Verburg 2019[23]** | **Zhao 2022[24]** | **Spargo 2019[28]** | **de Rooij 2022[30]** | **Escudero-Vilaplana 2020[31]** | **Mak 2016[32]** | **Li 2021[33]** | **Harman 2022[34]** | **Kampstra 2019[35]** | **Tejwani 2021[36]** | **Khaleva 2023[37]** |
| --- | --- | --- | --- | --- | --- | --- | --- | --- | --- | --- | --- | --- | --- | --- | --- | --- | --- | --- | --- | --- | --- | --- | --- | --- |
| **Scope specification** |  |  |  |  |  |  |  |  |  |  |  |  |  |  |  |  |  |  |  |  |  |  |  |  |
|  | 1 | The research or practice setting(s) in which the COS is to be applied | Y | Y | Y | Y | Y | Y | Y | Y | Y | Y | Y | Y | Y | Y | Y | Y | Y | Y | Y | Y | Y | Y |
|  | 2 | The health condition(s) covered by the COS | Y | Y | Y | Y | Y | Y | Y | Y | Y | Y | Y | Y | Y | Y | Y | Y | Y | Y | Y | Y | Y | Y |
|  | 3 | The population(s) covered by the COS | Y | Y | Y | Y | Y | Y | Y | Y | N | Y | N | Y | N | Y | N | Y | Y | N | N | N | Y | Y |
|  | 4 | The intervention(s) covered by the COS | N | N | Y | Y | N | N | N | Y | Y | Y | N | Y | Y | Y | Y | Y | Y | Y | Y | N | Y | Y |
| **Stakeholders involved** |  |  |  |  |  |  |  |  |  |  |  |  |  |  |  |  |  |  |  |  |  |  |  |  |
|  | 5 | Those who will use the COS in research | Y | Y | Y | Y | Y | Y | Y | Y | Y | Y | Y | Y | Y | Y | Y | Y | N | Y | Y | Y | Y | Y |
|  | 6 | Healthcare professionals with experience of patients with the condition | Y | Y | Y | Y | Y | Y | Y | Y | Y | Y | Y | Y | Y | Y | Y | Y | Y | Y | Y | Y | Y | Y |
|  | 7 | Patients with the condition or their representatives | Y | Y | N | Y | N | Y | Y | Y | Y | Y | Y | Y | Y | Y | Y | Y | Y | Y | Y | N | Y | Y |
| **Consensus process** |  |  |  |  |  |  |  |  |  |  |  |  |  |  |  |  |  |  |  |  |  |  |  |  |
|  | 8 | The initial list of outcomes considered both healthcare professionals' and patients' views | Y | Y | Y | Y | Y | Y | Y | Y | Y | Y | Y | Y | Y | Y | Y | Y | Y | Y | Y | Y | Y | Y |
|  | 9 | A scoring process and consensus definition were described a priori | N | N | Y | Y | N | Y | Y | Y | Y | Y | P | Y | Y | Y | Y | P | P | Y | Y | N | Y | P |
|  | 10 | Criteria for including/dropping/adding outcomes were described a priori | N | N | Y | Y | N | Y | Y | Y | Y | Y | P | Y | Y | Y | Y | Y | N | Y | Y | N | Y | N |
|  | 11 | Care was taken to avoid ambiguity of language used in the list of outcomes | N | N | N | Y | N | P | P | N | Y | Y | Y | N | P | Y | Y | N | N | P | Y | N | N | Y |

Note: N, no (=not reported); P, partly (=partially reported); Y, yes (= fully reported).
