# Supplementary material for: Assessment of the methodological quality of studies on core outcome sets for respiratory diseases: A systematic review and meta-research study
Source: PLoS One. 2025 Jan 2;20(1):e0316670. doi: 10.1371/journal.pone.0316670 (PMC11695018; doi:10.1371/journal.pone.0316670)
Supplement: S5 Table — (DOCX) [file pone.0316670.s005.docx]

**S5 Table. Compliance with COS-STAR Items.**

| **SECTION/TOPIC** | **ITEM No.** | **CHECKLIST ITEM** | **Tong 2020[11]** | **Tong 2021[12]** | **Jin 2020[13]** | **Qiu 2020[14]** | **Marshall 2020[15]** | **Munblit 2022[16]** | **Gorst 2023[17]** | **Shepherd 2022[18]** | **Souto-Miranda 2023[19]** | **Camus-García 2021[20]** | **Mathioudakis 2022[22]** | **Verburg 2019[23]** | **Zhao 2022[24]** | **Spargo 2019[28]** | **de Rooij 2022[30]** | **Escudero-Vilaplana 2020[31]** | **Mak 2016[32]** | **Li 2021[33]** | **Harman 2022[34]** | **Kampstra 2019[35]** | **Tejwani 2021[36]** | **Khaleva 2023[37]** |
| --- | --- | --- | --- | --- | --- | --- | --- | --- | --- | --- | --- | --- | --- | --- | --- | --- | --- | --- | --- | --- | --- | --- | --- | --- |
| **TITLE/**  **ABSTRACT** |  |  |  |  |  |  |  |  |  |  |  |  |  |  |  |  |  |  |  |  |  |  |  |  |
| Title | 1a | ldentify in the title that the paper reports the development of a COS | Y | Y | Y | Y | Y | Y | Y | Y | Y | Y | Y | Y | Y | Y | Y | Y | Y | Y | Y | Y | Y | Y |
| Abstract | 1b | Provide a structured summary | Y | Y | Y | Y | Y | Y | Y | Y | Y | Y | Y | Y | Y | Y | Y | Y | Y | Y | Y | Y | Y | Y |
| **INTRODUCTION** |  |  |  |  |  |  |  |  |  |  |  |  |  |  |  |  |  |  |  |  |  |  |  |  |
| Background and Objectives | 2a | Describe the background and explain the rationale for developing the COS | Y | Y | Y | Y | Y | Y | Y | Y | Y | Y | Y | Y | Y | Y | Y | Y | Y | Y | Y | Y | Y | Y |
|  | 2b | Describe the specific objectives with relerence to developing a COS | Y | Y | Y | Y | Y | Y | Y | Y | Y | Y | Y | Y | Y | Y | Y | Y | Y | Y | Y | Y | Y | Y |
| Scope | 3a | Describe the health condition(s) and population(s) covered by the COS | Y | Y | Y | Y | Y | Y | Y | Y | P | Y | P | Y | P | Y | P | Y | Y | P | P | P | Y | Y |
|  | 3b | Describe the intervention(s) covered by the COS | N | N | Y | Y | N | N | N | Y | Y | Y | N | Y | Y | Y | Y | Y | Y | Y | Y | N | Y | Y |
|  | 3c | Describe the setting(s) in which the COS is to be applied | Y | Y | Y | Y | Y | Y | Y | Y | Y | Y | Y | Y | Y | Y | Y | Y | Y | Y | Y | Y | Y | Y |
| **METHODS** |  |  |  |  |  |  |  |  |  |  |  |  |  |  |  |  |  |  |  |  |  |  |  |  |
| Protocol/Registry Entry | 4 | Indicate where the COS development protocol can be accessed, if available, and/or the study registration details | N | N | Y | Y | N | Y | Y | Y | Y | Y | Y | N | Y | Y | Y | N | N | Y | Y | N | N | Y |
| Participants | 5 | Describe the rationale for stakeholder groups involved in the COS development process, elgiblity criteria for participants from each group, and a description of how the individuals involved were identified | N | N | Y | P | N | Y | Y | Y | Y | Y | Y | P | N | N | N | N | N | N | N | Y | N | Y |
| Information Sources | 6a | Describe the information sources used to identify an initial list of outcomes | Y | Y | Y | Y | Y | Y | Y | Y | Y | Y | Y | Y | Y | Y | Y | Y | Y | Y | Y | Y | Y | Y |
|  | 6b | Describe how outcomes were dropped/combined, with reasons (if applicable) | N | N | Y | Y | N | N | Y | N | Y | P | P | N | Y | P | Y | N | N | Y | N | N | Y | N |
| Consensus Process | 7 | Describe how the consensus process was undertaken | Y | Y | Y | Y | Y | Y | Y | Y | Y | Y | Y | Y | Y | Y | Y | Y | Y | Y | Y | Y | Y | Y |
| Outcome Scoring | 8 | Describe how oulcomes were scored and how scores were summarised | N | N | Y | Y | N | Y | Y | Y | Y | Y | P | Y | Y | Y | Y | N | N | Y | Y | P | Y | N |
| Consensus Definition | 9a | Describe the consensus definition. | N | N | Y | Y | N | Y | Y | Y | Y | Y | Y | Y | Y | Y | Y | Y | Y | Y | Y | N | Y | Y |
|  | 9b | Describe the procedure for determining how outcomes were included or excluded from consideration during the consensus process | N | N | Y | Y | N | Y | Y | Y | Y | Y | N | Y | Y | Y | Y | Y | Y | Y | Y | N | Y | Y |
| Ethics and Consent | 10 | Provide a statement regarding the ethics and consent issues for the study | N | N | P | Y | N | P | Y | Y | Y | Y | N | Y | N | Y | Y | Y | N | Y | Y | N | N | Y |
| **RESULTS** |  |  |  |  |  |  |  |  |  |  |  |  |  |  |  |  |  |  |  |  |  |  |  |  |
| Protocol Deviations | 11 | Describe any changes from the protocol (if applicable), with reasons. and describe what impact these changes have on the results | N | N | N | N | N | Y | N | N | N | N | Y | N | N | N | Y | N | N | N | N | N | N | N |
| Participants | 12 | Present data on the number and relevant characteristics of the people involved at all stages of COS development | Y | Y | N | Y | N | Y | Y | Y | Y | Y | Y | P | Y | Y | Y | N | Y | Y | Y | Y | Y | Y |
| Outcomes | 13a | List all outcomes considered at the start of the consensus process | P | Y | Y | Y | Y | Y | Y | Y | Y | Y | Y | Y | Y | Y | Y | Y | N | Y | Y | Y | Y | Y |
|  | 13b | Describe any new outcomes introduced and any outcomes dropped, with reasons, during the consensus process | N | N | Y | Y | N | Y | Y | Y | Y | N | Y | Y | Y | Y | Y | Y | N | Y | Y | N | Y | Y |
| COS | 14 | List the outcomes in the final COS | Y | Y | Y | Y | Y | Y | Y | Y | Y | Y | Y | Y | Y | Y | Y | Y | Y | Y | Y | Y | Y | Y |
| **DISCUSSION** |  |  |  |  |  |  |  |  |  |  |  |  |  |  |  |  |  |  |  |  |  |  |  |  |
| Limitations | 15 | Discuss any limitations in the COS development process | N | Y | Y | Y | N | Y | Y | Y | Y | Y | Y | Y | Y | Y | Y | Y | Y | Y | Y | Y | Y | Y |
| Conclusions | 16 | Provide an interpretation of the final COS in the context of other evidence, and implications for future research | Y | Y | Y | Y | Y | Y | Y | Y | Y | Y | Y | Y | Y | Y | Y | Y | Y | Y | Y | Y | Y | Y |
| **OTHER INFORMATION** |  |  |  |  |  |  |  |  |  |  |  |  |  |  |  |  |  |  |  |  |  |  |  |  |
| Funding | 17 | Describe sources of funding/role of funders | Y | Y | Y | Y | Y | Y | Y | Y | Y | Y | Y | Y | N | Y | Y | Y | Y | Y | Y | Y | Y | Y |
| Conflicts of Interest | 18 | Describe any conflicts of interest within the study team and how these were managed | Y | Y | Y | Y | P | Y | Y | Y | N | N | Y | Y | N | Y | Y | Y | Y | N | Y | Y | Y | Y |

Note: N, no (=not reported); P, partly (=partially reported); Y, yes (= fully reported).
